# Quantifying potential leg axis alteration from extramedullary tibial guide malalignment in total knee arthroplasty: a computer simulation

**DOI:** 10.1186/s12891-026-10206-3

**Published:** 2026-07-17

**Authors:** Stefan M Zimmermann, Ken Bertschy, Christoph Zindel, Julian Hasler, Lukas Jud, Lazaros Vlachopoulos, Sandro F Fucentese

**Affiliations:** 1 Balgrist University Hospital, Forchstrasse 340, Zurich, 8008 Switzerland; 2 University of Zurich, Zurich, Switzerland

**Keywords:** Total knee arthroplasty, Extramedullary tibial alignment, Malalignment, Tibial slope, Mechanical axis

## Abstract

**Background:**

Despite technological advancements, the added benefit of navigated total knee arthroplasty has recently been questioned, and conventional instrumentation remains very common.

Rotational errors during tibial resection in total knee arthroplasty (TKA) may influence postoperative alignment. Although previous studies have shown changes in coronal projection under these conditions, the geometric relationship between axial rotation and the effective posterior tibial slope remains insufficiently defined.

The aim of this study was to quantify how axial malrotation of the tibial resection plane interacts with different posterior tibial slope targets and how these interactions affect both projected coronal alignment and the effective sagittal slope.

**Methods:**

Three-dimensional (3D) surface models of the lower extremity of 20 different patients were generated from computed tomography data. Tibial cuts were simulated, beginning with an osteotomy plane perpendicular to the tibial mechanical axis, which served as a baseline plane. For each predefined posterior tibial slope (3°, 5°, and 7°), axial malrotation of the tibial resection plane was systematically varied (± 5°, ± 10°, ± 20°, ± 40°), and the resulting changes in projected coronal alignment and effective posterior tibial slope were recorded.

**Results:**

Coronal deviation increased proportionally with both the magnitude of axial rotation and the planned posterior tibial slope. At a slope of 5°, ± 10° of malrotation resulted in a coronal deviation of 0.9° ± 0.01, increasing to ± 1.7° ± 0.01 at ± 20°. At a slope of 7°, ± 20° of rotation produced a coronal deviation of 2.4° ± 0.03. In contrast, the effective posterior tibial slope was minimally affected by axial rotation, changing by ≤ 0.3° even at ± 20° of malrotation.

**Conclusion:**

This study demonstrates that axial malrotation of a sloped tibial resection primarily affects the projected coronal mechanical alignment, while the effective posterior tibial slope itself remains largely preserved. The magnitude of coronal deviation increases with higher planned slope angles. In standard mechanically aligned scenarios, the isolated geometric effect appears limited; however, in slope-sensitive or individualized alignment strategies, rotational errors may contribute to measurable coronal projection changes.

## Introduction

Total knee arthroplasty (TKA) is the causative treatment for end-stage knee osteoarthritis and is currently the most common joint arthroplasty surgery worldwide [4], reliably providing pain relief and functional improvement [2, 5, 23]. Despite significant advancements in surgical techniques, implant design, and robotic assistance aimed at improving consistency, the rate of dissatisfied patients following TKA remains relatively high [6, 11, 14]. Robotic-assisted TKA (rTKA), though capable of reducing alignment outliers [22, 26], has not conclusively demonstrated superior clinical benefits over conventional methods [8, 13, 22], and its cost-effectiveness is still debated [17], which is why conventional instrumentation continues to be one of the most common methods for performing TKA today [1, 3].

In conventionally instrumented total knee arthroplasty, a tibial cutting guide is manually attached to an intra- or extramedullary tibial alignment rod to carry out the desired tibial cut. Although well described visual landmarks/ anatomical reference points exist to assist with correct attachment of the guide (trans-malleolar axis [9], medial third of tibial tubercle [29]), this manual fixation of the tibial alignment guide ultimately relies on eye judgement and may introduce errors leading to postoperative deviations in the mechanical leg axis.

While Tsukeoka et al. [27, 28] demonstrated that posterior slope amplifies coronal malalignment in the presence of rotational error, their analysis focused exclusively on coronal projection changes. The effect of axial malrotation on the effective posterior tibial slope itself, as well as the simultaneous geometric coupling between sagittal and coronal projections, has not been formally quantified. The present study extends prior work by providing a closed-form analytical framework that characterizes both coronal deviation and residual slope under rotational error [18].

The purpose of this study was not to predict the final postoperative mechanical alignment after total knee arthroplasty, but to quantify the isolated geometric sensitivity of the coronal and sagittal mechanical axes to axial rotational deviations of the tibial resection plane when using an extramedullary alignment guide.

Specifically, we aimed to determine how predefined axial malrotation angles interact with different posterior tibial slope targets and how these interactions translate into measurable changes in projected coronal and sagittal alignment.

We therefore hypothesized that (1) axial rotation of the tibial resection plane results in a predictable change in projected coronal alignment, (2) the magnitude of this effect increases with increasing posterior tibial slope, and (3) sagittal alignment remains largely unaffected by axial rotational errors.

## Materials & methods

The local ethical committee approved this study (Zurich Cantonal Ethics Commission, BASEC-Nr. 2023 − 00389), it was conducted in accordance with the principles of the Declaration of Helsinki. Informed consent was obtained from all patients. This research received no external funding. This was a descriptive laboratory study. 3D bone surface models of 20 lower extremities were generated from computed tomography (CT) data, with the knee in full extension. The models were sampled from a larger database of osteotomy cases to represent a range of anatomical geometries. As the aim of the study was to investigate a geometric relationship, sampling was performed independent of patient-specific clinical variables such as age, sex, BMI, or indication. CT imaging was performed using a SOMATOM Edge Plus scanner (Siemens Healthineers, Erlangen, Germany). Scans were acquired with a tube voltage of 100 kV, tube current of 350 mA, pitch 0.5, and a rotation time of 1.0 s. Images were reconstructed with a slice thickness of 1.0 mm using a 512 × 512 matrix, resulting in an in-plane resolution of 0.39 × 0.39 mm and a field of view of 200 mm. The scan range extended from the hip joint to the ankle joint, enabling assessment of the entire lower limb mechanical axis. Image analysis was performed using a standard bone window (W/L 1500/450) with multiplanar reconstructions.

The number of models was selected to represent anatomical variability across a range of tibial geometries rather than to achieve statistical power, given the deterministic geometric nature of the transformation. The anatomical models further served to validate the mathematical relationship within a 3D modelling environment and to facilitate a more intuitive understanding of the geometric effect across different anatomies.

**Fig. 1:**
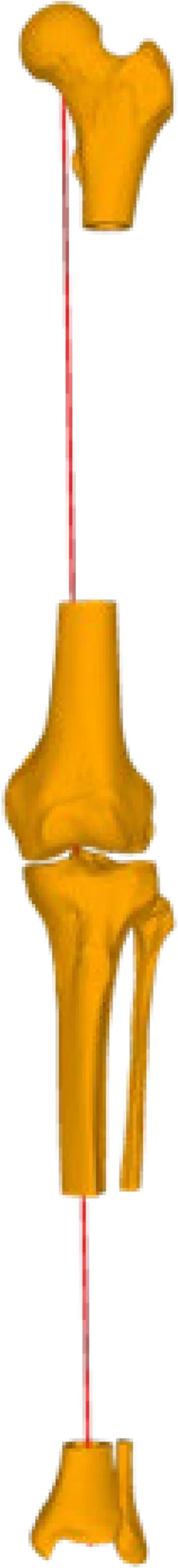
The mechanical axis of the limb is defined as the line connecting the center of the femoral head to the midpoint of the transmalleolar axis. To determine this axis, a sphere is fitted into the femoral head to identify its center. Similarly, spheres are placed over each malleolus to define their outer edges, and the centers of these spheres are connected to establish the transmalleolar axis

**Fig. 2:**
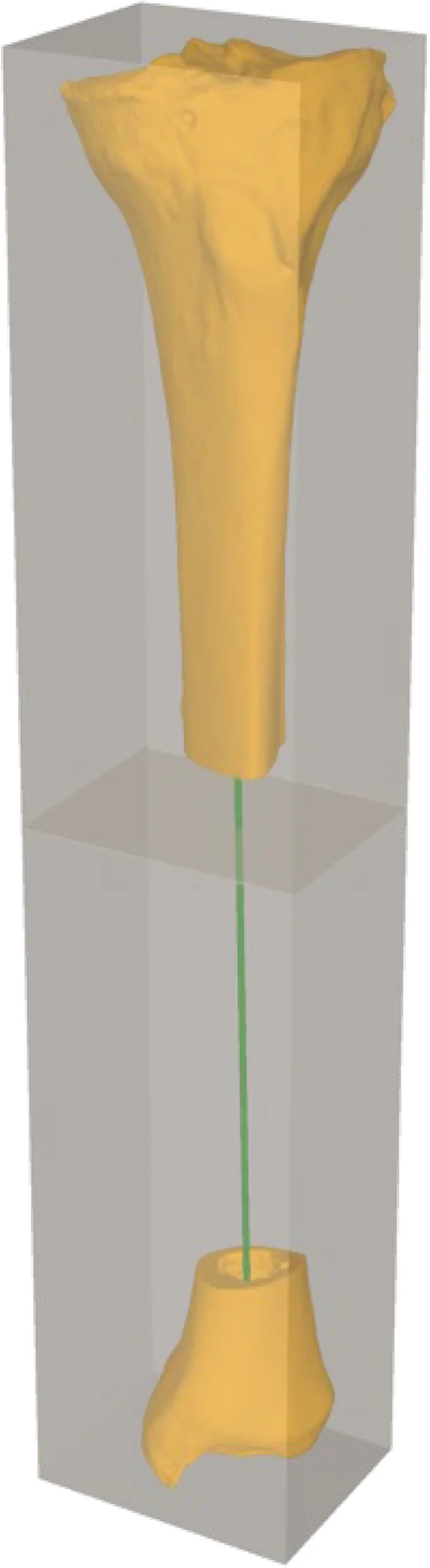
The green line illustrates the anatomical axis of the tibia, defined as a line passing through the center of the intramedullary canal. A reference cuboid was created to determine the full tibial length. Coronal cutting planes placed at 15% and 85% of this length to develop cross-sections of the intramedullary canal. The anatomical axis was then established by connecting the midpoints of these cross-sectional areas

Three-dimensional bone models of the femur, tibia, fibula, patella and talus were generated from CT data using commercial segmentation software (Mimics Medical 19.0, Materialise NV, Leuven, Belgium), with established accuracy for CT-based bone modelling. These models were imported into the CASPA surgical planning software Balgrist CARD AG, Zurich, Switzerland), where the mechanical axis was defined as described by Fürnstahl [10] (Figs. 1 and 2). Two cutting planes were generated at 15% and 85% of the tibial length to visualize the intramedullary canal area and to simulate the tibial osteotomy by creating surgical cutting planes aligned with the anatomical axis of the tibia.

**Fig. 3:**
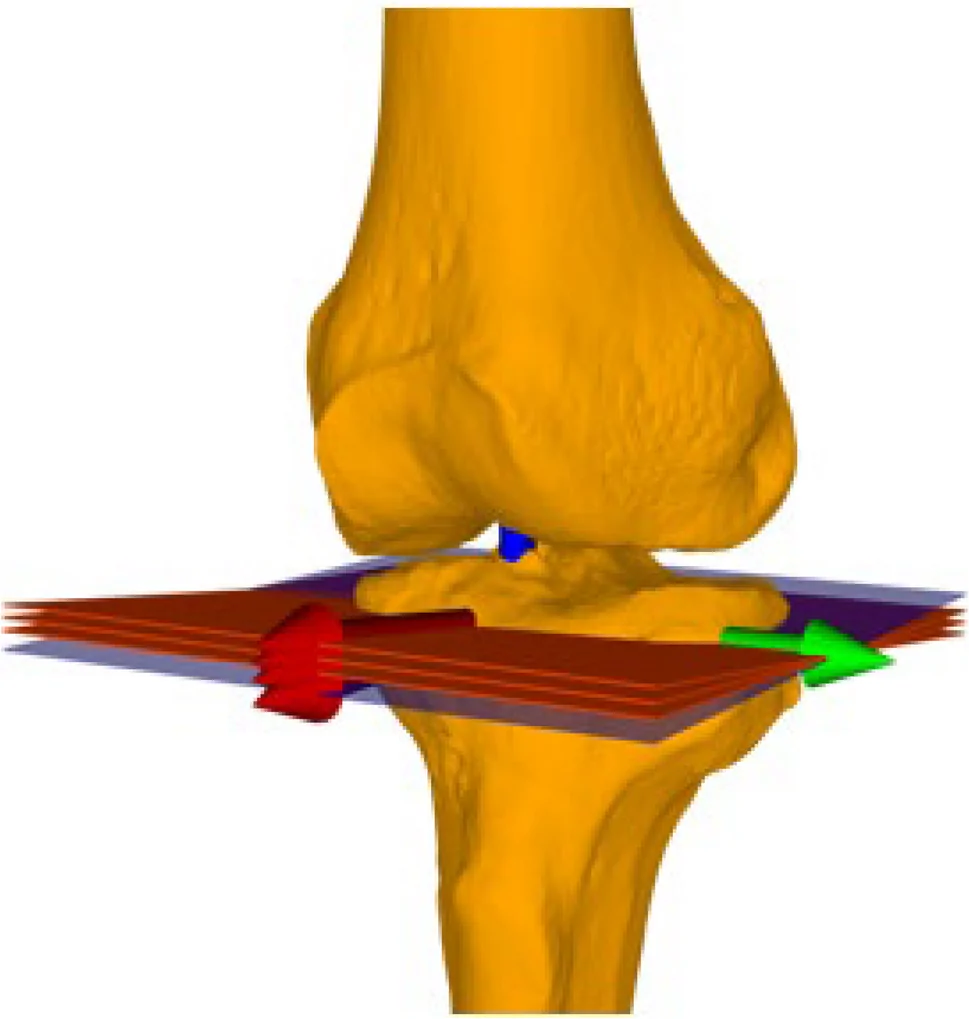
Bone model with three different tibial osteotomy planes with 3°, 5° and 7° of posterior tibial slope (blue plane = 0° slope)

As the targeted posterior tibial slope is usually 3° for a posterior stabilized component, between 5° and 7° for a medial pivot knee [24], and up to 7° for cruciate retaining components [7], respective cutting planes with 3°, 5°, and 7° of slope were generated to simulate different versions of the baseline cutting plane (Fig. 3).

**Fig. 4:**
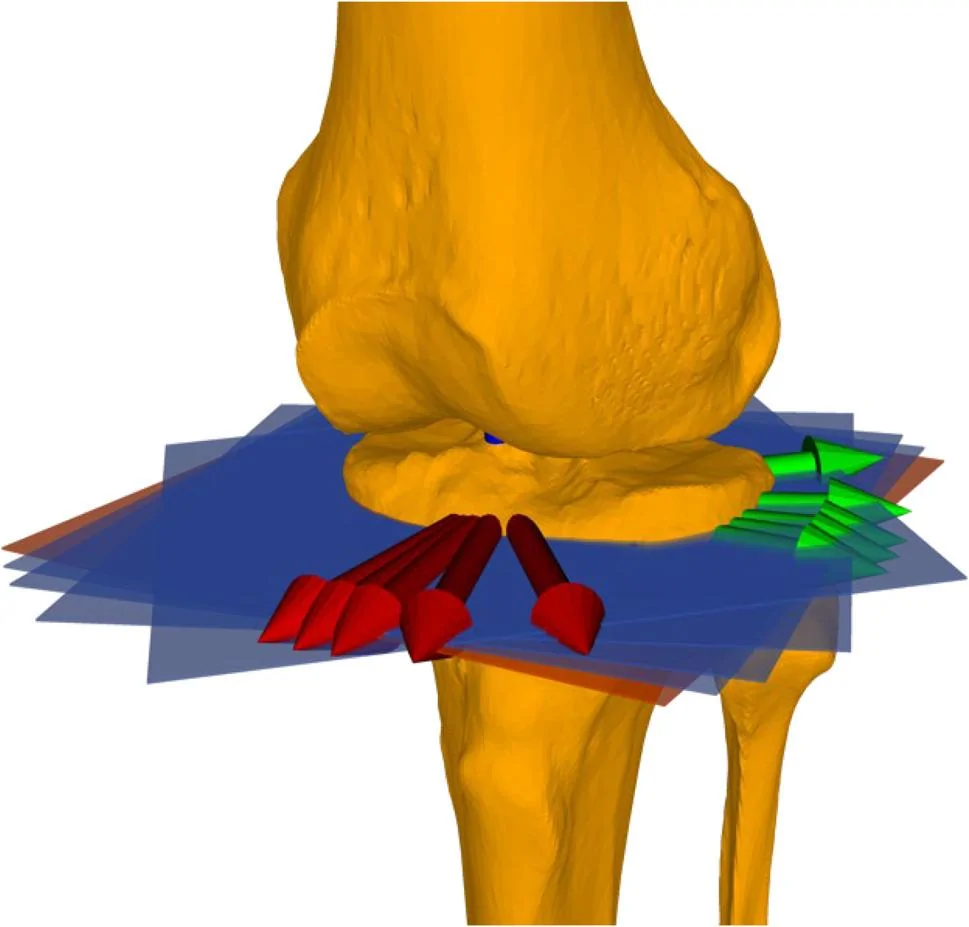
Bone model with four different tibial osteotomy planes with 5°, 10°, 20°, 40° of rotation as shown by the five red arrows (red plane with four rotational planes + orthogonal red arrow = 0° rotation)

For every tibial slope angle, a total of eight distinct osteotomies were generated, each involving internal rotations of the cutting plane at intervals of 5°, 10°, 20°, and 40°, as well as external rotations of -5°, -10°, -20°, and − 40° compared to a baseline cut oriented at the medial one third of the tibial tuberosity (Fig. 4).

Simulations were conducted for each osteotomy plane, and the corresponding deviation of the mechanical axis was measured [18].

In addition, a mathematical model was generated by an engineer (CZ) to compare the results of the computer simulation with a purely mathematical calculation of the problem. The mathematical model is based on a sequence of rotational vector calculations of the normal vector of the cutting plane. The final description includes the rotation around the coronal axis with the angle α (varus/valgus), around the sagittal axis with the angle β (slope) and around the axial axis with the angle γ (rotation).

Formula 1 shows the true influence of the malrotation (γ) by a planned slope (β) to the coronal leg alignment (α’, varus/valgus). Formula 2 shows the true remaining slope (β’) as influenced by the malrotation (γ) and the planned slope (β).

Formula 1: α’= arcsin (sin(β) * sin(γ))

Formula 2: β’= arcsin (sin(β) * cos(γ))

### Statistical analysis

Descriptive analysis was performed. Statistical calculation included mean values and standard deviation. The statistical analyses were carried out with SPSS for Windows (Version 29.0, SPSS inc, Chicago, IL, USA).

## Results

**Fig. 5:**
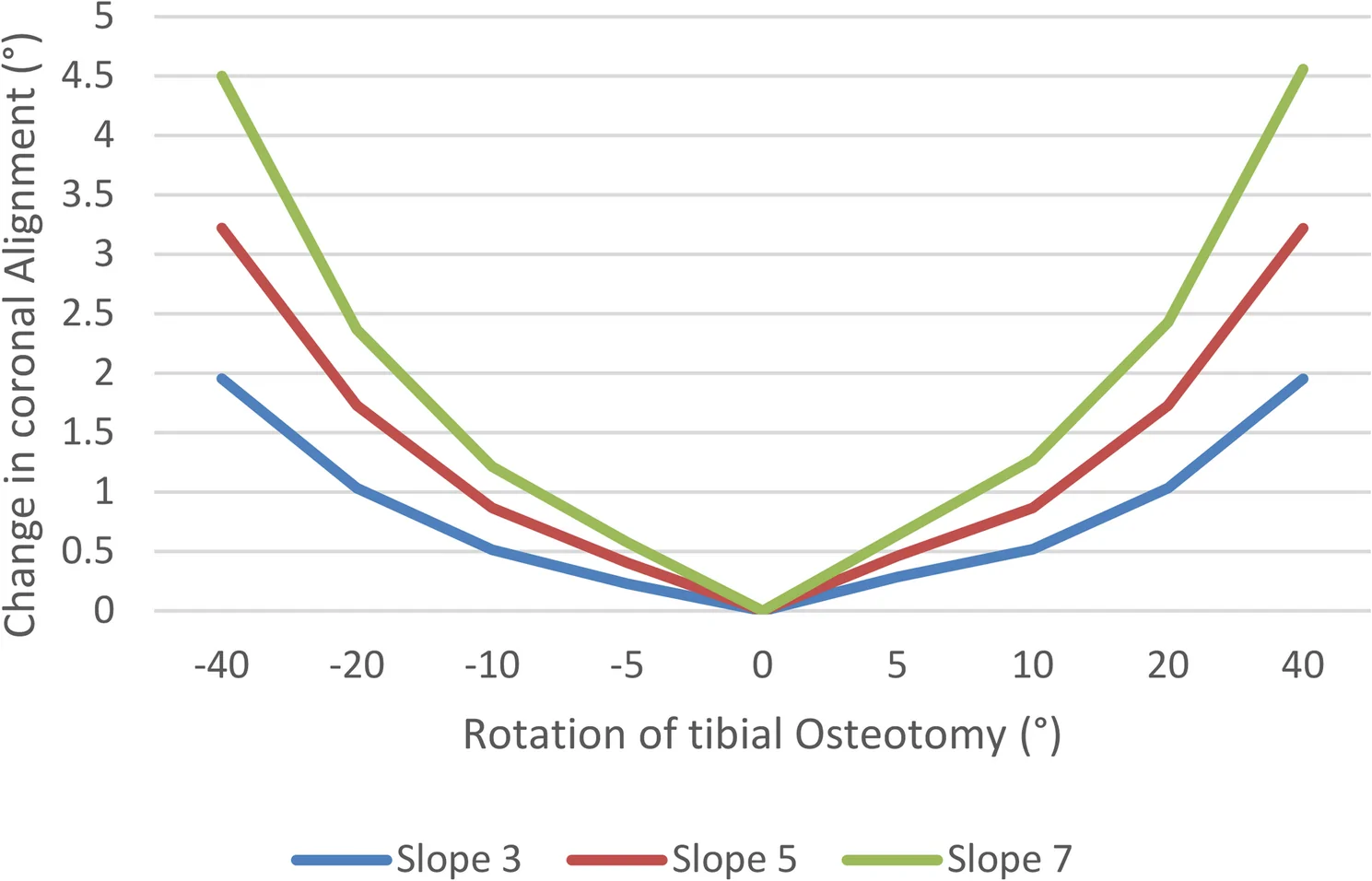
The effect of rotational error on coronal alignment

**Fig. 6:**
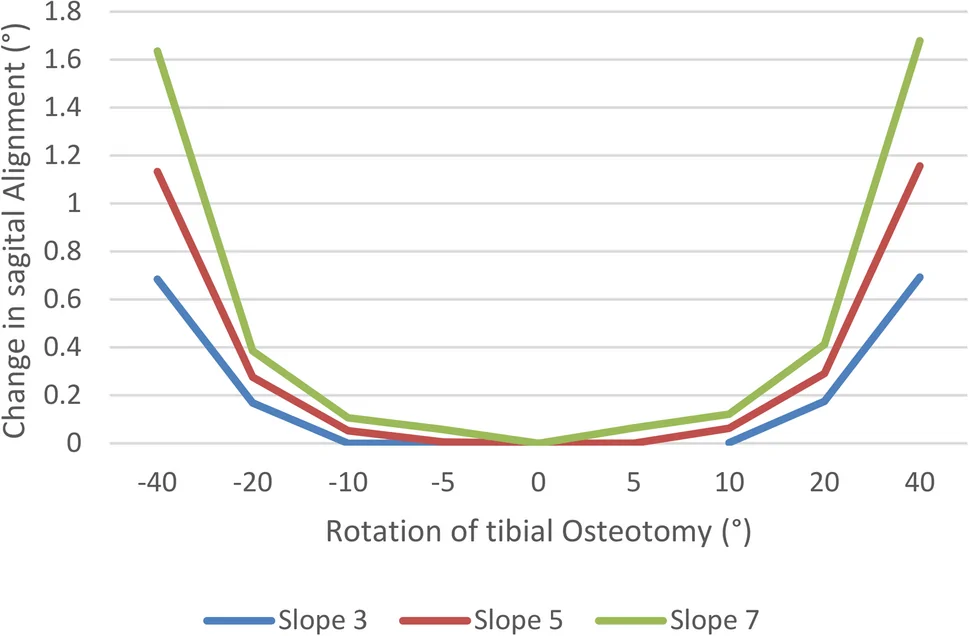
The effect of rotational error on sagittal alignment

The influence of axial rotation on coronal and sagittal mechanical alignment is summarized in Tables 1 and 2, and illustrated in Figs. 5 and 6. Generally, larger rotations and greater posterior slope angles led to increased deviations from the intended mechanical axis.

**Table 1:** The effect of rotational error on coronal alignment

Rotation	Mechanical Axis Deviation with Slope 3°	Mechanical Axis Deviation with Slope 5°	Mechanical Axis Deviation with Slope 7°
-40°	1.9	3.2	4.5
-20°	1.0	1.7	2.4
-10°	0.5	0.9	1.2
-5°	0.3	0.4	0.6
0°	0	0	0
5°	0.3	0.4	0.6
10°	0.5	0.9	1.2
20°	1.0	1.7	2.4
40°	1.9	3.2	4.5

**Table 2:** The effect of rotational error on sagittal alignment

Rotation	Mechanical Axis Deviation with Slope 3°	Mechanical Axis Deviation with Slope 5°	Mechanical Axis Deviation with Slope 7°
-40°	0.7	1.1	1.6
-20°	0.2	0.3	0.4
-10°	0	0.1	0.1
-5°	0	0	0.1
0°	0	0	0
5°	0	0	0.1
10°	0	0.1	0.1
20°	0.2	0.3	0.4
40°	0.7	1.2	1.6

### Coronal alignment

Rotational deviations up to ± 10° had minimal impact (< 2° change) at all slopes (3°, 5°, 7°).

At ± 20° rotation, deviations remained negligible (< 2°) for 3° and 5° slopes but became potentially relevant (2.4° ± 0.03) at a 7° slope.

At ± 40° rotation, deviations were minimal at 3° slope but clearly relevant at higher slopes (5° slope: ~3.2° ± 0.01; 7° slope: ~4.5° ± 0.02).

Simulations closely matched mathematical predictions, with differences ≤ 0.06°.

### Sagittal alignment

Axial rotation had negligible effects across all tested slopes, even at high rotational errors (± 20° produced ≤ 0.3° ± 0 change).

Mathematical and simulation data aligned closely (differences ≤ 0.03°).

Thus, axial rotational errors significantly impacted coronal alignment, especially when combined with higher tibial slopes, but had minimal effect on sagittal alignment.

## Discussion

### Key findings

The primary finding of this study is that axial malrotation of a sloped tibial resection plane predominantly affects the projected coronal mechanical alignment, whereas the effective posterior tibial slope remains largely preserved. The magnitude of coronal deviation increases proportionally with higher planned slope targets. Even moderate rotational errors therefore translate differently depending on the intended sagittal alignment strategy. These results quantify the geometric sensitivity of tibial alignment to rotational deviation under clinically used slope configurations.

While ongoing debate persists regarding the most favorable alignment philosophy in TKA, the overall objective remains consistent: achieving precise preoperative planning to ensure a stable knee joint with balanced ligamentous constraint. Various aids and technologies, from intra- and extramedullary alignment rods to robotic navigation, have been developed to facilitate accurate tibial and femoral cuts. This study focuses specifically on the tibial cut, investigating the amount of potential error associated with an extramedullary conventional alignment method. To this date, it remains one of the most common forms of instrumentation for the tibial cut. According to the latest Swiss National Joint Registry report, 70.6% of all TKA’s in Switzerland were performed with conventional instrumentation between 2016 and 2021 [25].

The selection of 3°, 5°, and 7° posterior tibial slope configurations covers the majority of clinically realistic scenarios. The rationale behind choosing eight different rotations of the osteotomy planes (5°/10°/20°/40°, both clock- and counter-clockwise) assumed of surgeons’ potential for errors during visual assessment of extramedullary tibial cutting guide attachment.

As such, it was assumed that errors of 5–10° in both internal- and external-rotation could realistically occur in clinical practice. The simulations of rotations of 40°, and arguably also 20°, are not realistic clinical scenarios, but were evaluated as well to illustrate the progression of such errors.

A key distinction from previous work lies in the bidirectional geometric analysis performed in the present study. Whereas prior investigations primarily reported coronal projection changes under combined slope and rotation configurations [27], we demonstrate analytically that axial malrotation has minimal influence on the effective posterior tibial slope itself. This differentiation between projected coronal deviation and preserved sagittal slope adds conceptual clarity and refines the biomechanical interpretation of rotational guide errors.

This additional dimension highlights that errors may become more relevant in cases that preoperatively plan to apply increased slope, such as in cruciate-retaining or medial-pivot designs. A further distinction is that the present analysis was reinforced by the use of a mathematical model based on rotational vector calculations, which closely reproduced the simulation results. This theoretical validation strengthens the reliability of our findings. Finally, graphical visualizations of the different cutting planes are included, illustrating how rotational errors translate into deviations of the mechanical leg axis.

### Clinical implications

In standard mechanically aligned TKA with moderate slope targets (3–5°), the isolated geometric contribution of tibial guide malrotation appears limited. Under these circumstances, conventional extramedullary alignment guides may provide sufficient geometric accuracy when established anatomical landmarks are carefully respected. However, in slope-sensitive scenarios with more extreme anatomy or aggressive alignment targets, such as cruciate-retaining implants with higher posterior slope or individualized alignment strategies including restricted or unrestricted kinematic alignment, the geometric amplification effect becomes more pronounced. In such cases, additional intraoperative verification of tibial guide orientation in both coronal and sagittal planes should be considered. Practical measures to minimize tibial guide malrotation include: (1) careful identification and consistent use of reliable anatomical landmarks, particularly the medial third of the tibial tuberosity and the ankle center; (2) simultaneous verification of guide alignment in both coronal and sagittal planes rather than relying on a single reference; and (3) consideration of navigation or robotic assistance in slope-sensitive or individualized alignment scenarios, especially when operating near predefined coronal alignment thresholds.

### Implications for contemporary alignment strategies

Contemporary alignment strategies in TKA differ substantially in their tolerance for coronal deviation and in their intended sagittal targets. In mechanically aligned TKA, tibial resection is typically performed perpendicular to the mechanical axis with moderate posterior slope, and small projection deviations may remain within widely accepted alignment corridors.

In contrast, kinematic and restricted kinematic alignment strategies intentionally aim for individualized coronal and sagittal targets, often including planned tibial varus and, in some cases, increased posterior slope. In these settings, the geometric amplification demonstrated in this study becomes more relevant, as axial malrotation may shift the projected coronal alignment beyond the intended personalized target. For instance: in a kinematically planned TKA with a targeted tibial varus of 5° and 7° slope, the overall varus within the tibia would end up at 6.2–6.3° ± 0.02 for ± 10° of malrotation and increase further to 7.4–7.5° ± 0.15 with 20° of malrotation, which surpasses the threshold typically deemed acceptable by most orthopedic surgeons, raising concerns about potential negative effects on joint biomechanics and long-term implant survivorship. However, it’s worth noting that acceptable extremes of the postoperative leg axis have been a subject of dispute in recent literature [16, 20].

Functional alignment concepts, which integrate patient-specific anatomy while maintaining defined safety boundaries, may also be sensitive to such projection effects when operating near predefined coronal thresholds.

The present findings therefore suggest that the geometric tolerance for tibial guide malrotation is not uniform across alignment philosophies but depends on the combination of planned slope and coronal target.

Previous studies have shown that rotational malpositioning of the extramedullary tibial alignment guide can influence the orientation of the tibial cut, particularly in the sagittal plane [21, 27]. While these foundational studies established the biomechanical relevance of rotational error, they primarily addressed the resulting sagittal deviation as a singular parameter and did not differentiate between overall sagittal alignment and posterior tibial slope. In contrast, the present study provides a detailed quantitative analysis of both components, with a specific focus on how varying posterior slope targets and modern alignment philosophies—such as kinematic or individualized alignment—may amplify the clinical impact of even minor rotational deviations. By modeling a wide range of realistic surgical scenarios, our findings offer contemporary relevance for preoperative planning and intraoperative execution in total knee arthroplasty.

### Limitations

This study has several limitations. The investigation represents a deterministic geometric sensitivity analysis and therefore isolates the mathematical effect of tibial resection plane rotation from biological and intraoperative variables. The model does not account for soft tissue balancing, femoral component positioning, bone quality, intraoperative loading conditions, or surgeon-related variability in visual guide placement. Consequently, while the mathematical validation confirms geometric consistency between simulation and analytical calculation, it does not establish clinical representativeness or predict postoperative patient outcomes. The present findings should therefore be interpreted as a quantification of geometric contribution rather than a comprehensive model of real-world surgical alignment.

Future research should integrate these geometric projections with clinical registry data or navigation-based intraoperative measurements to determine the real-world impact of rotational deviations on survivorship and functional outcomes.

Next, although only 20 CT-derived models were included to represent anatomical variability, the relationship between axial rotation and coronal projection follows a deterministic geometric principle. The very low dispersion of results (standard deviations in the range of 0.01–0.03°) reflects that the projected deviation is primarily determined by the rotational and slope parameters rather than by individual morphological differences. Within this cohort, inter-individual tibial morphology therefore had only minimal influence on the calculated projection changes. Nevertheless, the limited sample size may restrict generalizability, particularly to populations with substantially different limb geometries. As the analyzed models were derived from a European cohort, anatomical variations in other populations should be considered when extrapolating absolute alignment values, even though the underlying geometric projection mechanism remains consistent. Finally, inter- and intraobserver reliability (ICC) of the assessed radiological parameters were not explicitly evaluated as the reliability of the underlying measurements has been previously established in literature with near perfect agreement [12, 15, 19]. In addition, the majority of measurements in the present study were derived from a standardized, semi-automated 3D workflow, reducing observer dependency. Therefore, additional variability attributable to measurement inconsistency is expected to be minimal.

## Conclusion

This study quantifies the geometric relationship between axial malrotation of the tibial resection plane and resulting changes in coronal and sagittal mechanical alignment in conventionally instrumented TKA.

The analysis demonstrates that axial rotational errors increasingly influence coronal alignment as posterior tibial slope targets rise, whereas sagittal alignment remains minimally affected.

In standard mechanically aligned scenarios with moderate slope targets, the isolated geometric effect appears limited; however, in slope-sensitive or individualized alignment strategies, even moderate rotational deviations may contribute to measurable coronal projection changes.

These findings provide a quantitative framework for understanding the geometric sensitivity of tibial alignment techniques, while clinical validation in outcome-based studies remains necessary.

## Data Availability

The datasets generated and analyzed during the current study are available from the corresponding author on reasonable request.

## Abbreviations

3D: Three–dimensional
CASPA: Computer Assisted Surgical Planning Application
CT: Computed Tomography
OA: Osteoarthritis
PS: Posterior Stabilized
rTKA: Robotic–assisted Total Knee Arthroplasty
SIRIS: Swiss Implant Registry Information System
SPSS: Statistical Package for the Social Sciences
TKA: Total Knee Arthroplasty
